# A Multifaceted Presentation of Xerophthalmia in Autistic Patients

**DOI:** 10.7759/cureus.49172

**Published:** 2023-11-21

**Authors:** Annuar Zaki Azmi, Sylves Patrick, Mohamad Israk B Isa, Shuaibah Ab. Ghani

**Affiliations:** 1 Department of Paediatric Ophthalmology, Sabah Women and Children Hospital, Kota Kinabalu, MYS; 2 Department of Ophthalmology, Faculty of Medicine and Health Sciences, University Malaysia Sabah, Kota Kinabalu, MYS

**Keywords:** xerosis, keratomalacia, autism, xerophthalmia, vitamin a deficiency

## Abstract

We report the manifestations of vitamin A deficiency (VAD) in three children with underlying autism of different stages. These children were under developmental paediatrician follow-up for autism, and the VAD was not detected until these children presented to Ophthalmology screening for varying stages of signs and symptoms. On further assessment, all of our patients have VAD secondary to poor dietary intake, as autistic patients are associated with having selective eating habits. In our case series, we discuss the spectrum of xerophthalmia presentations, which can be mild and can manifest as punctate epithelial erosions to the more blinding complications at the advanced stage of the disease, mainly irreversible optic neuropathy. The primary management is to address the dietary routine coupled with systemic administration of vitamin A.

## Introduction

Vitamin A is an essential fat-soluble protein that plays multiple roles in our body. Vitamin A regulates the differentiation and proliferation of most cells in the human body [1]. In the eye, it maintains the integrity of mucosal cells. Children with underlying autism are prone to develop vitamin A deficiency (VAD) due to their selective eating [2]. Xerophthalmia is the spectrum of eye diseases caused by severe depletion of Vitamin A. One of the blinding complications of VAD is severe keratomalacia with potential corneal perforation [3]. Another blinding sequelae is optic neuropathy which is thought to be irreversible and signifying the end stage of the disease [4].

## Case presentation

Case 1

A seven-year-old boy with underlying mild autism complained to his parents that the child kept looking down with droopy eyelids for the past two weeks before the presentation. The father claimed that the child became more clumsy and watched television nearer than previously. Magnetic resonance imaging (MRI) was done, and no intracranial pathology was detected.

**Figure 1:**
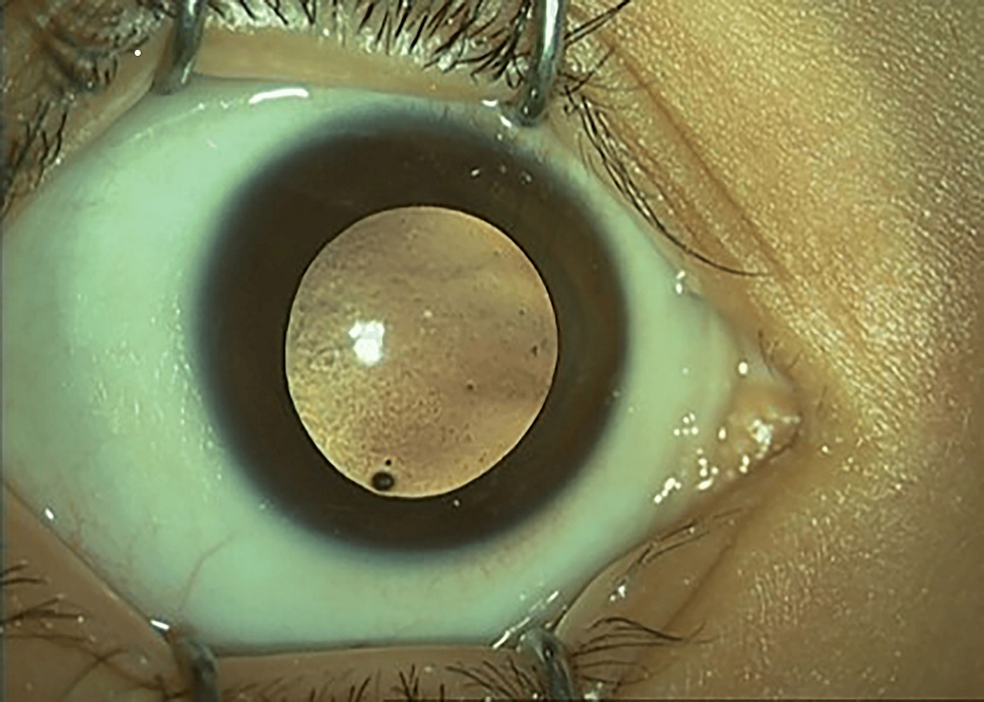
RE conjunctival xerosis RE: right eye

Visual acuity (VA) upon presentation was perception to light for both eyes (BE). Examination under anaesthesia revealed bilateral conjunctival xerosis and generalized punctate epithelial erosions (PEE) all over the cornea bilaterally but worst over the right eye (RE) (Figure 1). No Bitot's spot was seen. Fundus examination showed pale optic discs bilaterally. Investigation of serum vitamin A revealed a low vitamin A status of 0.12 umol/l (0.9-3.0 umo/l).

The child’s diet consisted of french fries thrice daily and junk food such as nuggets and chocolate. He also consumed plain porridge sometimes but refused meat and vegetables. He drinks formula milk twice a day. The patient was referred to a dietician, given systemic vitamin A therapy by the paediatrician and treated with frequent artificial tears. The child is keeping well with the resolvent of the corneal PEE and conjunctival xerosis. However, vision is poor with hand motion (HM) over the RE and 1/60 over the left eye (LE).

Case 2

A seven-year-old boy with autism presented with what was thought of as an LE perforated corneal ulcer upon presentation. The mother complained of LE redness intermittently for the past one month. There was no previous history of trauma. The best corrected visual acuity (BCVA) of RE was 6/12, and there was no light perception (NPL) for the LE.

**Figure 2:**
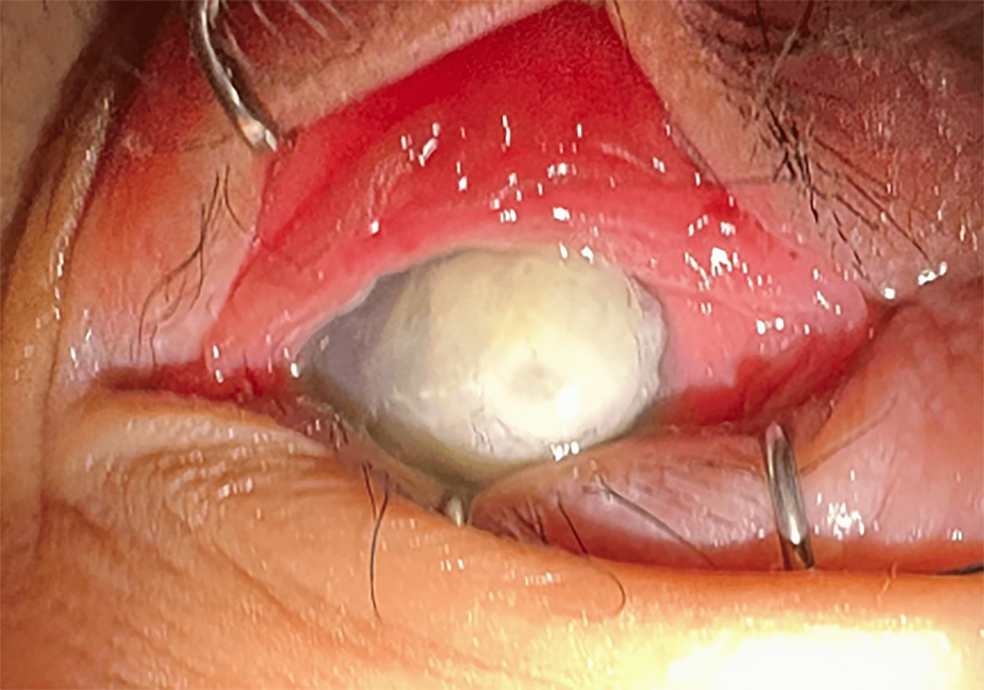
LE severe keratomalacia with perforated cornea LE: left eye

**Figure 3:**
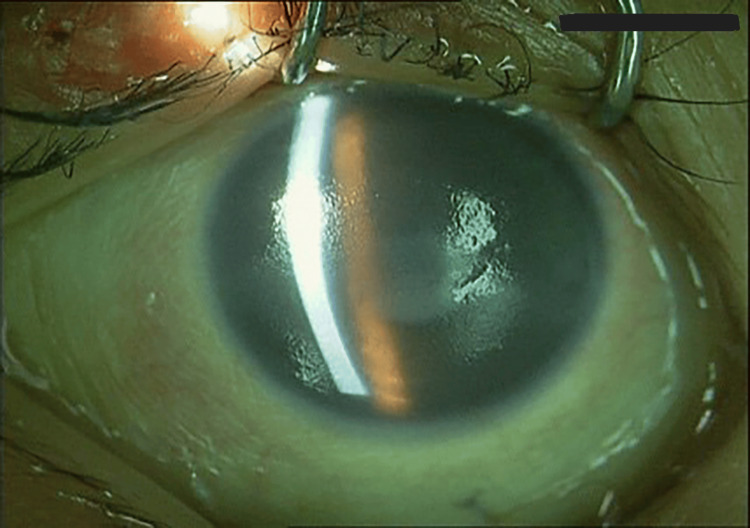
RE shows severe keratosis with generalized PEE. RE: right eye, PEE: punctate epithelial erosions

Upon examination, LE was shown to have severe leukomalacia with perforated cornea (Figure 2). RE shows severe keratosis with generalized PEE (Figure 3). Posterior segment examination of the RE shows a swollen optic disc; otherwise, the retina is flat. No view of the posterior segment of the LE. Serum vitamin A was taken and revealed a low vitamin A status of <0.10 umol/l (0.9-3.0 umo/l).

The child’s diet consisted of plain porridge for lunch and dinner and chocolate bars for breakfast (Kit Kat). He refused milk and only drank plain water and milo. The LE was unsalvageable and eventually had to be eviscerated. The patient was referred to a dietitian, and the paediatrician started systemic treatment of vitamin A. He was also given intensive topical artificial tears for RE. As of today, the RE cornea has only minimal PEE, but the conjunctival xerosis resolved. LE healed well following the evisceration.

Case 3

**Figure 4:**
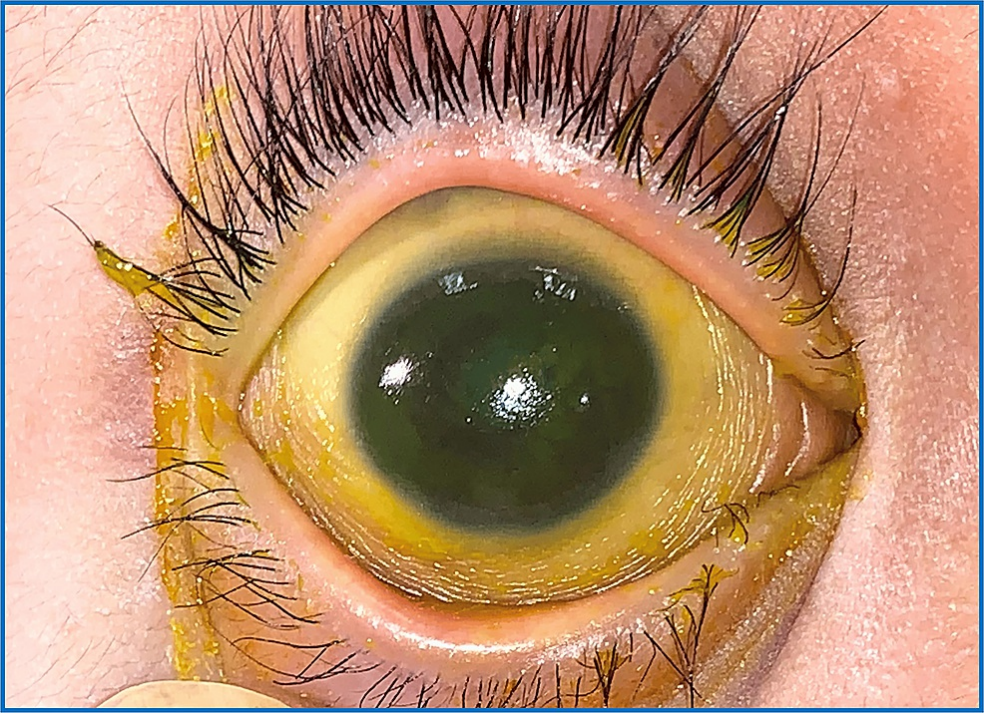
RE shows severe keratosis with generalized PEE. RE: right eye, PEE: punctate epithelial erosions

**Figure 5:**
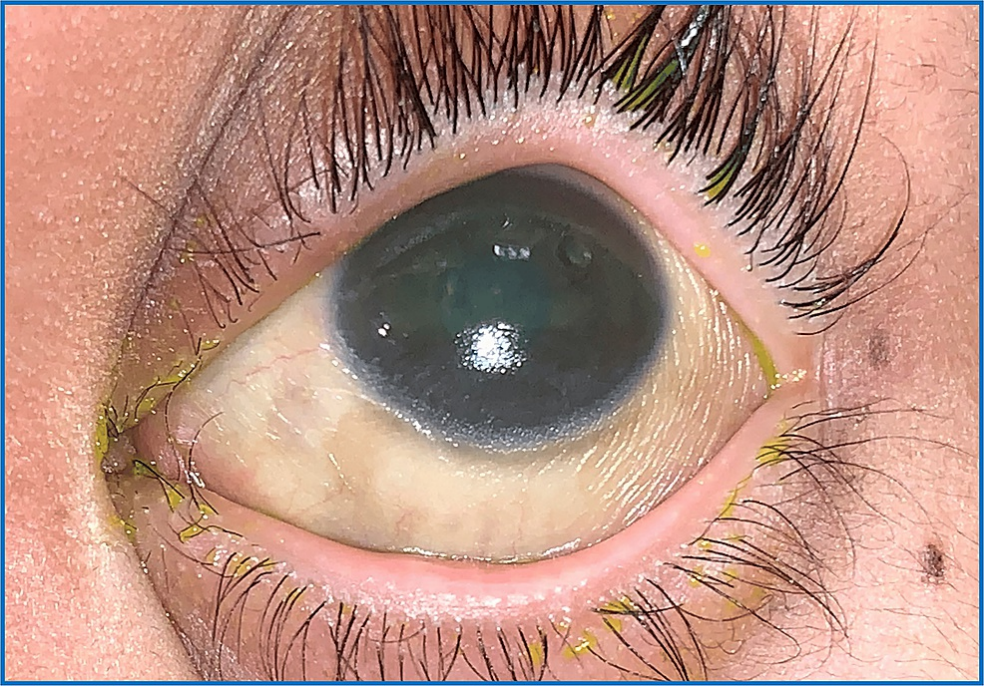
LE PEE and conjunctival xerosis LE: left eye, PEE: punctate epithelial erosions

A nine-year-old boy with underlying autism presented with bilateral eye redness on and off for the past two months prior to presentation. Upon examination, VA was 6/180 by using Lea Grating (uncooperative patient), and bilateral eye shows PEE and conjunctival xerosis but no Bitot's spot was noticed (Figures 4, 5). The posterior segment was otherwise unremarkable. Serum vitamin A revealed a low vitamin A status of <0.10 umol/l (0.9-3.0 umo/l). The patient was referred to a dietician and started systemic vitamin A therapy by the paediatrician. Intensive lubricants were also started.

Currently, the bilateral cornea is clear, with no conjunctival xerosis seen.

## Discussion

Vitamin A plays crucial roles in the eyes, specifically the conjunctiva, cornea and retina. 11-cis-retinal is the active form of vitamin A and is essential for the visual pathway. It allows electrochemical signalling to be generated from the light that falls onto the retina [5]. Vitamin A aids in the maintenance of the ocular surface and mucosal tissue integrity. Its presence is vital in tissue differentiation, particularly stratified squamous epithelium on the conjunctival surface [6]. Nutritionally, vitamin A can be obtained from dietary intake. Animal sources of vitamin A-rich food include dairy products, eggs, fish, and cod liver oil. They can also be found in vegetable produce such as spinach, lettuce, carrot and sweet potato [7].

Malaysia is currently at the level of mild subclinical VAD status. This study is based on a survey done by the Ministry of Health and UNICEF of more than 400 children under five years old and younger. Based on this survey alone, 4.5% of girls and 2.5% of boys had serum retinol levels ≤ 0.7 µmol/L [8]. Another local study was done using a semi-quantitative food frequency questionnaire (FFQ) to assess dietary intake, and the resulting nutrient intakes were then compared to Recommended Nutrient Intakes (RNI) by the Ministry of Health. The result shows that only 4.4% of Malaysian children between 6 months and 12 years old have low levels of vitamin A [9].

The presentation of vitamin A may be challenging to detect initially, especially in children with autistic disorders because the early manifestations usually start from the retina. It begins with photoreceptor dysfunction that manifests as night blindness or nyctalopia. As the vitamin A level gets low, the disease will start to affect the activity of the normal mucosal surface leading to their atrophy. This disease will present as Bitot’s spot appearing as a glistening surface caused by desquamated epithelium. As the disease progresses, the conjunctiva’s wrinkling, or conjunctival xerosis, will ensue, followed by loss of goblet cells [10].

All of our patients show the presence of conjunctival xerosis, which indicates that the disease has been there for some time - the second patient presented with severe leukomalacia leading to corneal perforation. To our knowledge, there is only one perforation case in VAD with autism secondary to severe corneal ulcer [3]. The second patient shows optic nerve swelling for the third case, indicating that optic nerve involvement is possible, albeit rare. Optic atrophy is another optic nerve involvement indicating end-stage disease with poor visual prognosis [4].

Autistic children are associated with selective diets with decreased protein, vegetables, fruits and even dairy intakes [10]. Some studies also show that they have preferentiality towards a starch diet [11]. Nutritional progress should be monitored in autistic patients, especially those with selective intakes. It is known that VAD is becoming scarcely rare in the developed world, but caution should be looked upon for autistic children with picky eating. On top of the developmental assessment, they should be considered for vitamin A serum level testing and prompt ophthalmology screening from the onset of diagnosis [6].

The principal treatment for VAD is to correct the deficiency and manage the eye manifestations [12]. For children with a developmental disorder such as autism, a holistic approach is needed to address its developmental aspect and any nutritional deficiencies to prevent sinister sequelae that may be initially undetected. In the presence of behavioural and visual changes, one should consider serum testing of vitamin levels. The input of a dietician is valuable to help autistic children cope with their changing dietary needs once the child passes the acute stages of disease manifestations [11].

## Conclusions

Children with autism have a high index of suspicions towards VAD and other nutrients due to their tendency for selective eating. A routine examination should be co-managed with a developmental paediatrician and dietician. Nutritional aspects for autistic patients should be explored from the get-go to prevent sinister complications, as mentioned. A baseline of vitamin A should be taken with periodic testing of vitamin A in a patient with dietary issues until the child establishes an acceptable dietary routine.
